# Spontaneously Hypertensive Rats (SHR) Are Resistant to a Reserpine-Induced Progressive Model of Parkinson’s Disease: Differences in Motor Behavior, Tyrosine Hydroxylase and α-Synuclein Expression

**DOI:** 10.3389/fnagi.2017.00078

**Published:** 2017-03-27

**Authors:** Anderson H. F. F. Leão, Ywlliane S. R. Meurer, Anatildes F. da Silva, André M. Medeiros, Clarissa L. C. Campêlo, Vanessa C. Abílio, Rovena C. G. K. Engelberth, Jeferson S. Cavalcante, Geison S. Izídio, Alessandra M. Ribeiro, Regina H. Silva

**Affiliations:** ^1^Memory Studies Laboratory, Department of Physiology, Federal University of Rio Grande do NorteNatal, Brazil; ^2^Brain Institute, Federal University of Rio Grande do NorteNatal, Brazil; ^3^Behavioral Neuroscience Laboratory, Department of Pharmacology, Federal University of São PauloSão Paulo, Brazil; ^4^Department of Pharmacology, Federal University of São PauloSão Paulo, Brazil; ^5^Neurochemical Studies Laboratory, Department of Physiology, Federal University of Rio Grande do NorteNatal, Brazil; ^6^Laboratory of Behavioral Genetics, Department of Cellular Biology, Embryology and Genetics, Federal University of Santa CatarinaFlorianopolis, Brazil; ^7^Department of Biosciences, Federal University of São PauloSantos, Brazil

**Keywords:** reserpine, Parkinson’s disease, SHR, α-synuclein, tyrosine hydroxylase

## Abstract

Reserpine is an irreversible inhibitor of vesicular monoamine transporter-2 (VMAT2) used to study Parkinson’s disease (PD) and screening for antiparkinsonian treatments in rodents. Recently, the repeated treatment with a low-dose of reserpine was proposed as a progressive model of PD. Rats under this treatment show progressive catalepsy behavior, oral movements and spontaneous motor activity decrement. In parallel, compared to Wistar rats, spontaneously hypertensive rats (SHR) are resistant to acute reserpine-induced oral dyskinesia. We aimed to assess whether SHR would present differential susceptibility to repeated reserpine-induced deficits in the progressive model of PD. Male Wistar and SHR rats were administered 15 subcutaneously (s.c.) injections of reserpine (0.1 mg/kg) or vehicle, every other day and motor activity was assessed by the catalepsy, oral movements and open field tests. Only reserpine-treated Wistar rats presented increased latency to step down in the catalepsy test and impaired spontaneous activity in the open field. On the other hand, there was an increase in oral movements in both reserpine-treated strains, although with reduced magnitude and latency to instauration in SHR. After a 15-day withdrawn period, both strains recovered from motor impairment, but SHR animals expressed reduced latencies to reach control levels. Finally, we performed immunohistochemistry for tyrosine hydroxylase (TH) and α-synuclein (α-syn) 48 h after the last injection or 15 days after withdrawn. Reserpine-treated animals presented a reduction in TH and an increase in α-syn immunoreactivity in the substantia nigra and dorsal striatum (dSTR), which were both recovered after 15 days of withdraw. Furthermore, SHR rats were resistant to reserpine-induced TH decrement in the substantia nigra, and presented reduced immunoreactivity to α-syn in the dSTR relative to Wistar rats, irrespective of treatment. This effect was accompanied by increase of malondaldhyde (MDA) in the striatum of reserpine-treated Wistar rats, while SHR presented reduced MDA in both control and reserpine conditions relative to Wistar strain. In conclusion, the current results show that SHR are resilient to motor and neurochemical impairments induced by the repeated low-dose reserpine protocol. These findings indicate that the neurochemical, molecular and genetic differences in the SHR strain are potential relevant targets to the study of susceptibility to PD.

## Introduction

Parkinson’s disease (PD) is the second most prevalent neurodegenerative disease, estimated to affect 1%–2% of those older than 60 years, and 7–10 million people worldwide (Mayeux, 2003; Van Den Eeden et al., 2003). Most importantly, it is a disorder with progressive onset and escalating deterioration of life quality (Braak et al., 2003). Therefore, the resulting social and economic burdens to countries with increasing life expectancy justify a growing scientific effort to understand its physiopathology.

The disease is characterized by motor impairments including muscular weakness, rigidity, postural instability, tremors and bradykinesia (Klockgether, 2004; Duty and Jenner, 2011). However, non-motor alterations such as anxiety, depression, anhedonia, deficit in executive function, dementia and sleep disturbances are also present (Comella, 2003; Schneider et al., 2003; Bowers et al., 2006; Ishihara and Brayne, 2006; Barone, 2011; Gómez-Esteban et al., 2011; Voon and Dalley, 2011). The motor symptoms are the result of the progressive and irreversible loss of dopaminergic neurons in the substantia nigra pars compacta (SNpc). Such loss of dopaminergic inputs concurrently progresses with the accumulation of intracellular α-synuclein (α-syn)-rich protein inclusions, known as Lewy bodies (McNaught et al., 2001; Lotharius and Brundin, 2002).

The scientific literature highlights a number of genes associated to the inheritance of PD, such as *α-syn*, *parkin*, *PINK1*, *DJ-1* and *LRRK*. Nevertheless, familial forms of PD explains only 10%–15% of the cases (Gao and Hong, 2011). The majority of cases are reported as idiopathic or sporadic, and are associated to environmental factors such as exposition to pesticides, rural life, infection, head trauma, and emotional disturbances like anxiety and depression (Godeiro et al., 2010; Noyce et al., 2012). Nonetheless, these epidemiological findings suggest that there are cumulative interactions between genetic and environmental factors that lead to the disease (Kim et al., 2005).

Despite many studies investigating the relevance of punctual genetic mutations in neurotoxic PD animal models (von Bohlen und Halbach et al., 2004; von Bohlen und Halbach, 2006), few of them investigated the differences between strains upon the expression of the parkinsonian phenotype. The use of distinct rodent strains could help to enlighten mechanisms related to sensitivity to neurotoxic insults. Specifically, physiological variations related to susceptibility to a dopaminergic insult in different rat strains would be relevant in the context of the multifactorial etiology of PD. For example, a study associated the resistance to the dopaminergic insult induced by the parkinsonian agent 1-methyl-4-phenyl-1,2,3,6-tetrahydropyridine (MPTP) to autosomic alleles of the C57BL/J6 mice strain (Hamre et al., 1999). Of notice, spontaneously hypertensive rats (SHR) are resistant to the high-dose reserpine-induced oral dyskinesia. Further, this resistance is attributed to the increased activity of the antioxidant enzyme catalase in the striatum exhibited by the SHR animals (Queiroz et al., 1998; Abílio et al., 2004). Interestingly, studies have shown that SHR present other neurochemical differences compared to normotensive strains that could be related to the pathophysiology of PD, such as variances in dopaminergic systems (Viggiano et al., 2004) and decreased α-syn expression in the hippocampus (Chiavegatto et al., 2009). However the effects of parkinsonian-inducing agents on these parameters have not been investigated in this strain yet.

Reserpine is a vesicular monoamine transporter-2 (VMAT2) inhibitor extracted from *Rauwolfia serpentina*. This drug depletes monoamines in the central nervous system and produces lethargy, depression and dyskinesia (Leão et al., 2015). Due to these properties, reserpine was employed to model the motor and non-motor deficits related to PD in rodents and screen for candidate pharmacological treatments to PD (Jurna et al., 1973; Goldstein et al., 1975; Ossowska et al., 1994; Fernandes et al., 2012; Santos et al., 2013; Leão et al., 2015). Briefly, the reserpine model recapitulates substantial features of symptomatology, neurochemistry and pharmacology of the disease (for a review see Leão et al., 2015). Recently, we have proposed a repeated low-dose reserpine treatment that produces progressive motor and non-motor symptoms of PD, oxidative damage to membrane lipids, monoamine depletion and reduction in tyrosine hydroxylase (TH) in the nigrostriatal pathway (Fernandes et al., 2012; Santos et al., 2013; Leão et al., 2015). In view of Abílio et al.’s (2004) findings, which were focused on high-dose acute reserpine-induced dyskinesia, we predict that SHR animals would also be more resistant to repeated low-dose reserpine-induced progressive motor impairment and cellular alterations. We suggest that the comparison between SHR and Wistar rats under this treatment could provide relevant information to the understanding of susceptibility to factors involved in PD etiology. Thus, we aimed to compare the development of motor impairments and cellular hallmarks of PD (expression of TH and α-syn, and membrane lipid peroxidation) between SHR and Wistar rats using the repeated low-dose reserpine protocol.

## Materials and Methods

### Animals

We used 7-month-old male Wistar and SHR rats. All animals were housed in groups of 4–5 per cage (30 cm × 37 cm × 16 cm) under conditions of acoustic isolation and controlled airflow and temperature (23 ± 1°C), with a 12 h light/12 h dark cycle (lights on 6:30 a.m.). Food and water were available *ad libitum*. Animals used in this study were handled in accordance with the guidelines of the Brazilian law for the use of animals in research (Law Number 11.794). All procedures were approved by the local ethics committee (Comissao de Etica no uso de animais da Universidade Federal do Rio Grande do Norte, CEUA-UFRN #005/2014, and Comissao de Etica no uso de animais da Universidade Federal de Sao Paul, CEUA-UNIFESP #9239070815) and all efforts were made to minimize animal pain, suffering or discomfort.

### Drugs and General Procedures

Reserpine (Sigma Chemical Co., St. Louis, MO, USA) was dissolved in two drops of glacial acetic acid and diluted in distilled water (0.1 mg/ml). Vehicle consisted of the same amount of acetic acid and water as in the reserpine solution. These solutions were injected subcutaneously (s.c.). We briefly handled animals for 2 min during 3 days before the beginning of the experimental procedures.

### Experimental Design

**Figure 1:**
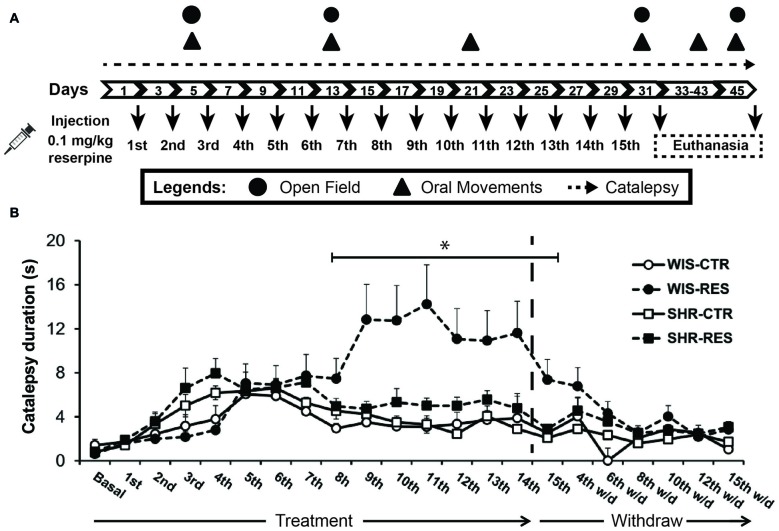
**(A)** Schematic representation of the experimental design. **(B)** Effects of repeated administration of 0.1 mg/kg reserpine on catalepsy behavior in Wistar and spontaneously hypertensive rats (SHR) rats. Data are expressed as mean ± SEM relative to WIS-CTR group. *p < 0.05 comparing WIS-RES to WIS-CTR (Two-way analysis of variance (ANOVA) with repeated measures followed by Sidak’s *post hoc* test).

The rats from each strain (Wistar and SHR) were randomly assigned to one of three groups: control (WIS-CTR: *n* = 8; SHR-CTR: *n* = 10), reserpine-treated (WIS-RESt: *n* = 8; SHR-RESt: *n* = 10) and reserpine-withdrawn (WIS-RESw/d: *n* = 8; SHR-RESw/d: *n* = 10) groups. The animals received 15 s.c injections of vehicle (CTR) or 0.1 mg/kg of reserpine (RESt and RESw/d) at a volume of 1 ml/kg body weight, every other day. The rats of the RESt group were euthanized 48 h after the 15th injection, while CTR and RESw/d animals were euthanized 15 days after the 15th injection, for immunohistochemmical procedures. Motor behavior data from RESt and RESw/d were considered together for analysis until RESt group was euthanized (RES group). Rats went through the following behavioral procedures (from 8:00 h to 16:00 h): (1) catalepsy test before the 1st injection and every other day (8:00–9:00 h) throughout the treatment and for 15 days after the last injection; (2) evaluation of spontaneous activity in the open field after the 2nd, 6th and 15th injections, and 15 days after withdraw (9:00–12:00 h); and (3) oral movements quantification after the 2nd, 6th, 10th and 15th injection, and 10 and 15 days after withdraw (13:00–16:00 h). The behavioral tests listed were performed ~48 h after the referred injection, before the animals received the following injection (16:00–17:00 h). Experimental design is shown in Figure 1A.

### Motor Behavior

#### Catalepsy Test

The catalepsy behavior was assessed by placing the animal’s forepaws on a horizontal bar positioned at 9 cm above the bench surface. Catalepsy was defined as an immobile posture, keeping both forepaws on the bar. The duration of catalepsy was measured up to a maximum of 180 s. Three trials were carried out for each animal in each observation day and the mean value of the three trials was considered for analysis.

#### Spontaneous Activity in the Open Field

The apparatus was a circular open field arena (84 cm in diameter) with 32 cm high walls, made of wood and covered in black laminated plastic. Animals were placed in the center of the apparatus for free exploration during 5 min. A digital camera above the apparatus recorded the sessions and an animal video-tracking software (ANY-maze, Stoelting, Wood Dale, IL, USA) registered the behavioral parameters. We quantified the distance traveled (in meters) and the time moving above the speed of 0.05 m/s in the apparatus (in seconds).

#### Oral Movements

Rats were individually placed in a wired cage (40 cm × 40.5 cm × 20 cm). Mirrors were positioned under the floor and behind the back wall of the cage to allow behavioral quantification when the animal faced away from the observer. The number of tongue protrusions (projection of the tongue out of the oral cavity) and the frequency of vacuous chewing movements (mouth openings in the vertical plane not directed toward physical material) were measured continuously for 10 min.

### TH and α-syn Immunohistochemistry

Upon completion of the behavioral procedures, all animals were anesthetized with an intraperitoneal injection of sodium thiopental (40 mg/kg) and perfused transcardially with 200 ml phosphate-buffered saline (PBS), pH 7.4, containing 500 IU heparin (Liquemin, Roche, Brazil), followed by 300 ml 4.0% paraformaldehyde in 0.1 M phosphate buffer, pH 7.4. The brains were removed from the skull, postfixed in the same fixative solution for 2–4 h, and transferred to a solution containing sucrose 30% in 0.1 M PBS, pH 7.4. Each brain was serially cut in the coronal plane into 50-μm thick sections with a cryostat microtome (Leica, Germany) at a temperature of −20°C. The sections were placed sequentially in five compartments (one section per compartment, 250-μm apart) and stored in antifreeze solution. Free-floating sections were incubated for 18–24 h with a polyclonal anti-TH (cat # AB152 Chemicon, USA, 1:10,000, as described by Santos et al. (2013) or anti-α-syn (cat # sc-7011-R, Santa Cruz Inc., Santa Cruz, CA, USA, 1:500) primary antibody raised in rabbit, containing 2% goat normal serum diluted in 0.3% Triton X-100 and 0.1 M phosphate buffer, pH 7.4. Sections were incubated with the biotinylated secondary antibody anti-rabbit (1:1000; cat # S-1000 Vector Labs, Burlingame, CA, USA) obtained in goat for 2 h at room temperature, washed, and incubated with avidin–biotin-peroxidase solution (ABC Elite kit, Vector Labs, Burlingame, CA, USA) for 90 min. The reaction was developed by the addition of diaminobenzidine tetrahydrochloride (Sigma, NY, USA) and 0.01% H_2_O_2_ in 0.1 M phosphate buffer, pH 7.4. The sections were washed (4×, 5 min) with 0.1 M phosphate buffer, pH 7.4, between each step and at the end of the procedure. Then, the sections were dried, dehydrated in a graded alcohol series, cleared in xylene, and cover slipped with Entellan (Merck). The immunostainings were performed in four sessions with balanced number of animals per group in each session, minimizing possible differences in background between the groups due to small variations in immunostaining. Sections were examined under brightfield illumination (Olympus Microscope, BX-41), images were captured using a CCD camera (Nikon, DXM-1200) and the locations of areas were determined using the atlas of Paxinos and Watson (2009).

### Immunohistochemistry Quantification

In order to estimate the number of dopaminergic cells in the SNpc four sections of each animal were selected: one at the rostral level, two at the medium level and one at the caudal level, representative of the rostrocaudal extension of SNpc. The exact location of the region was determined on the basis of the Paxinos and Watson rat brain atlas (2009; AP: −5.04; −5.40; −5.52; −6.00 relative to the bregma). Briefly, using an automated plate microscope (Olympus Microscope, BX-41) and the software Stereo Investigator (MBF Bioscience, Williston, VT, USA) we delineated the SNpc bilaterally in the coronal sections previously described and sampled the number of TH+ neuron bodies from 75 × 75 (μm) frames into a 200 × 200 (μm) grid under a 100× oil immersion objective. The TH+ cell count was performed for the whole extension within each section. Number of neuron count was then expressed relative to WIS-CTR group (Santos et al., 2013).

Additionally, TH and α-syn levels were assessed by analysis of relative optical densitometry (ROD) in the dorsal striatum (dSTR; AP: 1.08; 0.36; 0.00; −0.72 relative to bregma) and substantia nigra pars reticulada (SNpr, in the same coronal sections as for the SNpc) using the software ImageJ (Version 1.46i, NIH). α-syn expresses in synaptic terminals, and a previous study has shown immunoreaction for this protein in the SNpr, co-localized with inhibitory terminals from the dSTR. Of notice, that study did not show any protein inclusion, fibrils or neuron body staining in the SNpc (Taguchi et al., 2016). The SNpr receives abundant afferences from striatal medium spiny neurons and is one of the two output nuclei from the basal ganglia to the motor thalamus, working as a fast-spiking pacemaker in the absence of synaptic input (DeLong and Wichmann, 2007; Da Cunha et al., 2009). Thus, the quantification of α-syn expression in the SNpr may provide some understanding on the status of the indirect pathway in the basal ganglia.

Four representative sections of the rostrocaudal extension of each region were chosen. In each section, five fields evenly distributed throughout the areas of interest and control region were analyzed. The medium pixels in the target area were subtracted from the medium values of a control region (areas that should not have specific TH staining) of the same tissue (cortex or corpus calosum). Finally, all values were normalized considering the Wistar control group, in order to evaluate proportional alterations (Santos et al., 2013).

### Lipid Peroxidation Assay

We performed an experiment to assess membrane lipid peroxidation after reserpine treatment. We administered the repeated-reserpine treatment protocol (15 s.c. injections of 0.1 mg/kg, every other day) to Wistar and SHR (WIS-CTR = 7; WIS-RES = 7; SHR-CTR = 8; SHR-RES = 8). Forty-eight hours after the last injection animals were euthanized by decapitation, the brains were removed and the striatum were dissected billateraly. The tissue was immediately weighed and homogenized in phosphate buffer 0.1 M (1:5). Samples were centrifuged for 15 min at 3500 rpm and 4–5°C and duplicates of the supernatant of each sample were used in the reaction. Lipid peroxidation was estimated by the quantification of malondialdehyde (MDA)—a fluorescent product formed from the reaction of this aldehyde with thiobarbituric acid (TBA), as described by Tanizawa et al. (1981). The reaction was initiated by the addition of 250 μL of SDS 3%, 1.5 ml of acetic acid buffer 2M, and 1.5 ml of TBA 0.8% to 50 μL of the tissue homogenate. The volume was completed with 4 ml of ultrapure water, incubated in a water bath at 95°C for 60 min and cooled in an ice-cold water bath. Finally, 2.5 ml from a n-butanol:pyridine (15:1) mixture was added and samples were vigorously agitated for 30 s. The results were expressed in nanomoles (nm) of MDA per gram (g) of wet tissue calculated by plotting the obtained fluorescence (excitation at 315 nm, emission at 553 nm) against a standard MDA concentration curve.

### Data Analysis

Kolmogorov-Smirnov’s and Levene’s tests were used to analyze normality of data and homogeneity of variance, respectively. Parametric tests were used accordingly to data distribution and homogeneity of variance. Two-way analysis of variance (ANOVA) with repeated measures—with treatment and strain as between-subject factors and sessions as within-subject factor—were applied to catalepsy, open field and oral movement motor parameters to assess effects throughout treatment and withdraw phases. A two-way ANOVA was applied to immunohistochemistry parameters with treatment and strain as between-subject factors. Both tests were followed by Sidak’s *post hoc* test to highlight differences between strain and treatment groups. Results were expressed as mean + SEM and *p* < 0.05 was considered to reflect significant differences. Exact *p*-values were expressed for each factor and factor interactions for the two-way ANOVA, while differences highlighted by the Sidak’s *post hoc* test are assumed *p* < 0.05.

## Results

### Motor Behavior

#### Catalepsy Test

Two-way ANOVA with repeated measures revealed effects of session (*F*_(15,750)_ = 3.187; *p* < 0.001), session*strain (*F*_(15,750)_ = 2.766; *p* < 0.001), session*treatment interaction (*F*_(15,750)_ = 3.162; *p* < 0.001), time*treatment*strain interaction (*F*_(15,750)_ = 1.950; *p* = 0.016) and treatment (*F*_(1,50)_ = 4.151); *p* = 0.047) during the treatment phase. The same analysis to the withdraw phase did not reveal significant effects, and pointed out only a marginal effect of time (*F*_(6,192)_ = 2.120); *p* = 0.053). Sidak’s *post hoc* test yielded differences only to WIS-RES group, with animals spending more time in the bar than any other group from 48 h after the 7th injection (day 17) to 48 h after the 15th injection (day 31) of reserpine (Figure 1B). That is, the repeated administration of reserpine resulted in the progressive increase of latency to step down the bar only in the Wistar strain, and this motor impairment was restored after treatment withdrawn (Figure 1B).

#### Spontaneous Locomotion in the Open Field

##### Total distance traveled

**Figure 2:**
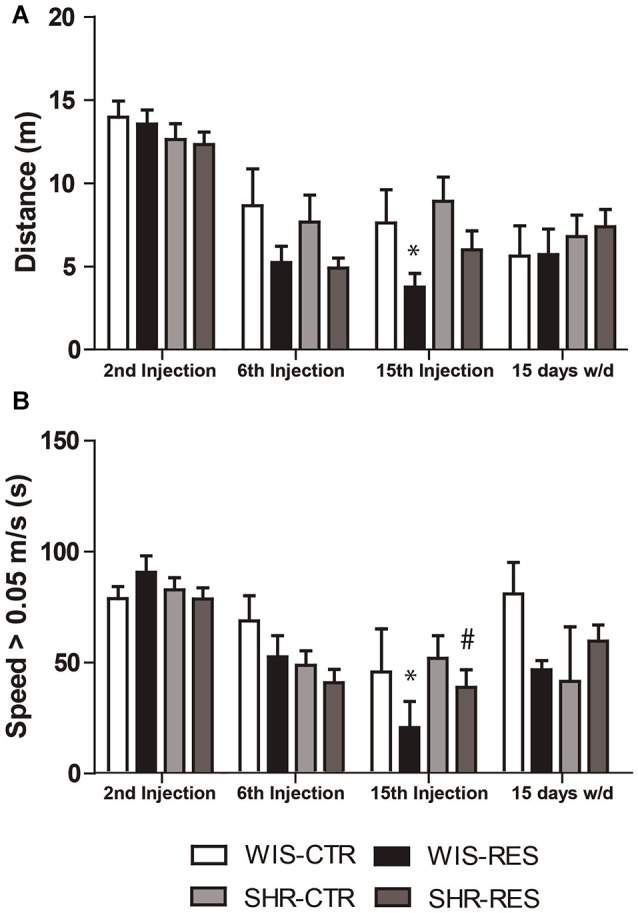
**Effects of repeated administration of 0.1 mg/kg reserpine on (A)** total distance traveled and **(B)** time in speed above 0.05 m/s. Data are expressed as mean ± SEM relative to WIS-CTR group. *p < 0.05 compared to respective CTR group; ^#^*p* < 0.05 compared to respective treatment in WIS strain (Two-way ANOVA with repeated measures followed by Sidak’s *post hoc* test).

Two-way ANOVA with repeated measures in the treatment phase revealed effects of treatment (*F*_(1,50)_ = 6.18; *p* = 0.016), session (*F*_(2,100)_ = 69.17; *p* < 0.001), session*treatment interaction (*F*_(2,100)_ = 3.37; *p* = 0.038) and session*strain interaction (*F*_(2,100)_ = 3.21; *p* = 0.044). Two-way ANOVA did not found significant effects in the withdraw phase. Differences between groups in each time point were yielded by Sidak’s *post hoc* test and shown in Figure 2A. Only WIS-RES group traveled a shorter distance relative to WIS-CTR group 48 h after the 15th injection.

##### Speed above 0.05 m/s

Two-way ANOVA with repeated measures in the treatment phase revealed effects of session (*F*_(2,100)_ = 29.26; *p* < 0.001) and session*strain interaction (*F*_(2,100)_ = 3.08; *p* = 0.050). Two-way ANOVA did not found significant effects in the withdraw phase. Differences between groups in each time point were yielded by Sidak’s *post hoc* test and shown in Figure 2B. Briefly, only WIS-RES group spent less time moving above 0.05 m/s relative to WIS-CTR and SHR-RES groups 48 h after the 15th injection.

Taken together, open field data indicate that the repeated administration of reserpine resulted in the progressive impairment of spontaneous motor activity in both strains, but with higher magnitude for the Wistar strain. Also, motor impairment was restored after treatment withdrawn.

#### Oral Movements

##### Vacuous chewing

**Figure 3:**
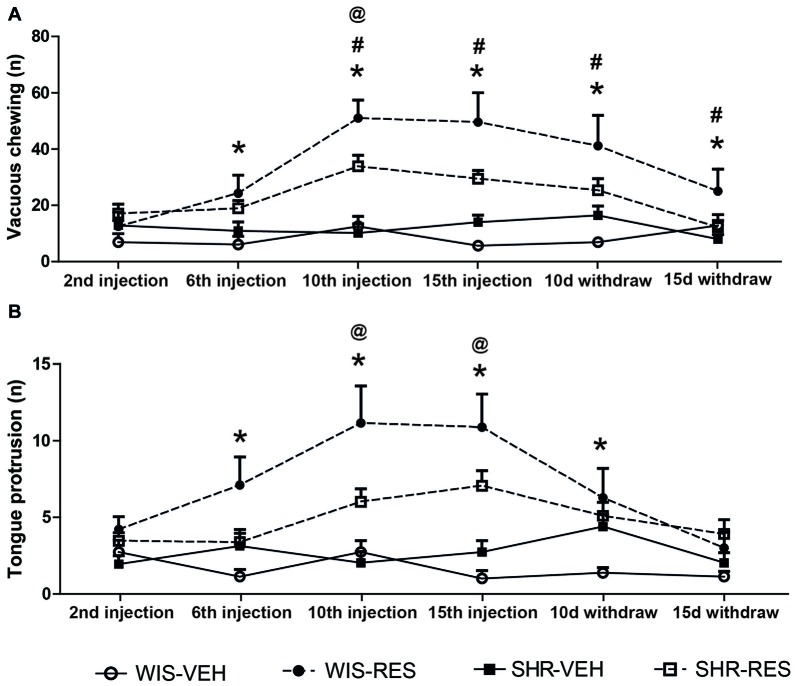
**Effects of repeated administration of 0.1 mg/kg reserpine on (A)** vacuous chewing movements, and **(B)** tongue protrusions in oral movement test in Wistar and SHR rats. Data are expressed as mean ± SEM. *p < 0.05 WIS-RES compared to WIS-CTR; ^@^*p* < 0.05 SHR-RES compared to SHR-CTR; ^#^*p* < 0.05 WIS-RES compared to SHR-RES (Two-way ANOVA with repeated measures followed by Sidak’s *post hoc* test).

Two-way ANOVA with repeated measures in the treatment phase revealed effects of session (*F*_(3,150)_ = 13.81; *p* < 0.001), treatment (*F*_(1,50)_ = 23.33; *p* < 0.001), session*strain interaction (*F*_(3,150)_ = 3.16; *p* = 0.026) and session*treatment interaction (*F*_(3,150)_ = 11.33; *p* < 0.001). The same analysis revealed effect of session (*F*_(1,32)_ = 12.56; *p* < 0.001), treatment (*F*_(1,32)_ = 12.56; *p* = 0.001), session*treatment*strain interaction (*F*_(1,32)_ = 4.83; *p* = 0.035) and strain*treatment interaction (*F*_(1,32)_ = 4.42; *p* = 0.043) in the withdraw phase. Differences between groups in each time point were yielded by Sidak’s *post hoc* test and shown in Figure 3A. WIS-RES presented increase in the vacuous chewing movements relative to respective strain control from the 6th injection to 10 days after withdraw, while SHR-RES presented increase in vacuous chewing only after the 10th injection. As well, differences in the magnitude of increase in vacuous chewing between reserpine-treated strains occurred from the 10th injection to 15 days after withdraw, with WIS-RES presenting higher values than SHR-RES group.

##### Tongue protrusion

Two-way ANOVA with repeated measures in the treatment phase revealed effects of session (*F*_(3,150)_ = 5.15; *p* < 0.05), treatment (*F*_(1,50)_ = 18.59; *p* < 0.001) and session*treatment interaction (*F*_(3,150)_ = 5.94; *p* < 0.001). The same analysis revealed effect of time (*F*_(1,32)_ = 8.17; *p* < 0.05) and treatment (*F*_(1,32)_ = 8.48; *p* < 0.05) in the withdraw phase. Differences between groups in each time point were yielded by Sidak’s *post hoc* test and shown in Figure 3B. WIS-RES groups presented increase in the tongue protrusion relative to WIS-CTR from the 6th injection to 10 days after withdraw, while SHR-RES (relative to SHR-CTR) presented increased values only after the 10th and 15th injections. No differences in the magnitude of increase in tongue protrusion between reserpine-treated strains was detected.

Overall, repeated reserpine treatment resulted in the progressive increase in oral movements in both strains, but with higher magnitude in the Wistar strain. Nevertheless, this effect upon oral movements was restored after treatment withdrawn.

### Neurochemistry

#### Tyrosine Hydroxylase

**Figure 4:**
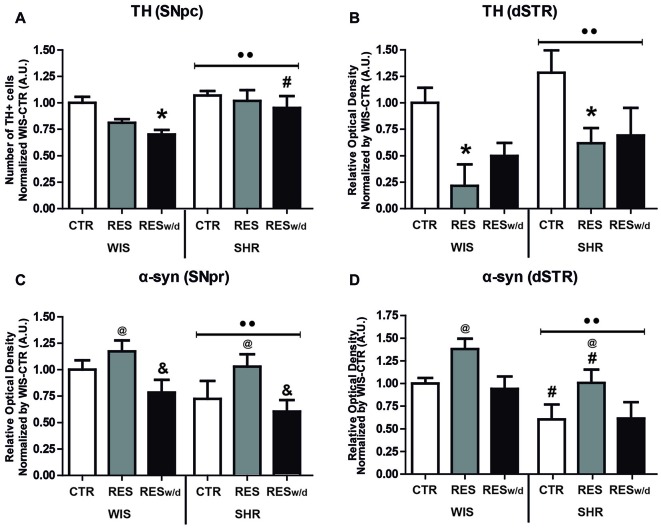
**Effects of repeated administration of 0.1 mg/kg reserpine on immunostaining for tyrosine hydroxylase (TH) in the (A)** substantia nigra pars compacta (SNpc) and **(B)** dorsal striatum (dSTR), and α-synuclein (α-syn) in the **(C)** SNpc and **(D)** dSTR in Wistar and SHR rats. Data are expressed as mean ± SEM relative to WIS-CTR group. *p < 0.05 compared to respective CTR group; ^#^*p* < 0.05 compared to respective treatment in WIS strain; ^&^*p* < 0.05 compared to respective RESt group; ^••^*p* < 0.05 effect of strain; ^@^*p* < 0.05 effect of treatment (Two-way ANOVA followed by Sidak’s *post hoc* test).

Two-way ANOVA for the number of TH positive neurons in the SNpc revealed effects of treatment (*F*_(2,54)_ = 3.39; *p* = 0.041) and strain (*F*_(1,54)_ = 7.44; *p* = 0.009; Figure 4A). Sidak’s *post hoc* test revealed that WIS-RESw/d group presented reduction in the number of TH+ neurons in the SNpc relative to WIS-CTR. Moreover, SHR-RESt and SHR-RESw/d groups did not present any differences in the TH+ neuron count compared to SHR-CTR. Further, SHR-RESw/d displayed increased TH+ neuron count in the SNpc compared to WIS-RESw/d group. That is, only WIS rats presented reductions in TH expression in the SNpc after reserpine treatment.

Two-way ANOVA for TH expression in the dSTR revealed only effects of treatment (*F*_(2,54)_ = 7.61; *p* = 0.001), but a marginal effect for strain (*F*_(1,54)_ = 2.48; *p* = 0.068) was pointed out (Figure 4B). Both WIS-RESt and SHR-RESt groups presented a reduction in TH expression in the dSTR compared to respective CTR groups. Despite this tendency, RESw/d groups did not differ from CTR groups in any strain. Nevertheless, they did not differ from RESt groups either. Differences between groups revealed by Sidak’s *post hoc* test are shown in Figures 4A,B.

Overall, reserpine treatment resulted in a reduced immunoreactivity to TH in the nigrostriatal pathway, but SHR animals appeared to be more resistant to such decrease.

#### α-Synuclein

α-syn expression in the SNpc did not reveal any protein inclusion, fibrils or neuron body staining. Two-way ANOVA for α-syn expression in the SNpr revealed effects of treatment (*F*_(2,54)_ = 5.48; *p* = 0.007) and strain (*F*_(1,54)_ = 4.27; *p* = 0.045). Sidak’s *post hoc* test revealed that the SHR-RESt group presented increased immunoreactivity to α-syn in the SNpr compared to the SHR-RESw/d group (Figure 4C).

Two-way ANOVA for α-syn expression in the dSTR revealed effects of treatment (*F*_(2,54)_ = 5.39; *p* = 0.008) and strain (*F*_(1,54)_ = 9.59; *p* = 0.003). Both RESt groups presented an increase in α-syn immunostaining compared to respective CTR and RESw groups. Importantly, SHR-CTR and SHR-RESt group presented a reduction of expression compared to respective treatment groups in the WIS strain. Differences between groups revealed by Sidak’s *post hoc* test and shown in Figure 4D.

Overall, reserpine treatment increased α-syn immunostaining, and this effect was restored after withdraw. Of notice, SHR strain displayed reduced α-syn expression regardless of treatment.

**Figure 5:**
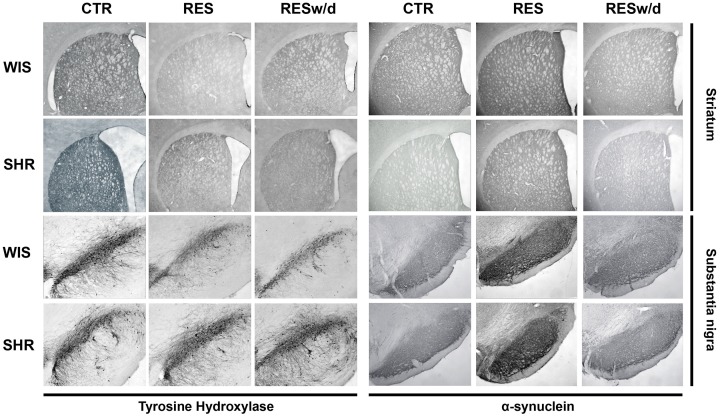
**Representative photomicrographs of brain coronal sections of dSTR and substantia nigra of rats repeatedly treated with vehicle (CTR) or 0.1 mg/kg reserpine euthanized 48 h after last injection (RESt) or 15 days (RESw/d) after the last injection.** Scale bar in dSTR: 1000 μm; and substantia nigra: 200 μm.

Representative sections of each region analyzed for TH and α-syn immunohistochemistry are presented in Figure 5.

#### Lipid Peroxidation Assay

**Figure 6:**
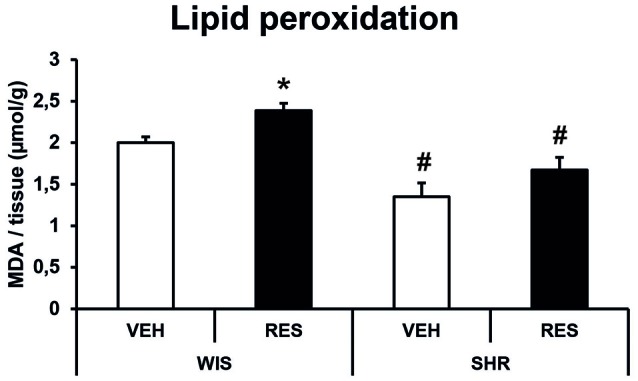
**Effects of repeated administration of 0.1 mg/kg reserpine on lipid peroxidation (malondyaldehyde—MDA formation) in the striatum of Wistar and SHR rats.** Data are expressed as mean ± SEM. *p < 0.05 compared to respective CTR group; ^#^*p* < 0.05 compared to respective treatment in WIS strain (Two-way ANOVA followed by Sidak’s *post hoc* test).

Two-way ANOVA for MDA quantification revealed effects of treatment (*F*_(1,28)_ = 7.817; *p* = 0.01) and strain (*F*_(1,28)_ = 28.536; *p* < 0.001; Figure 6). Sidak’s *post hoc* test revealed that WIS-RES group presented increased lipid peroxidation in the striatum compared to WIS-CTR. Moreover, SHR-CTR and SHR-RES groups presented decreased MDA concentration in the striatum compared to the respective treatment group in Wistar strain. Nonetheless, SHR-CTR and SHR-RES were not different from each other (Figure 6). That is, SHR strain presented reduced basal levels of MDA compared to Wistar, and reserpine-treatment induced an increase in lipid peroxidation only in the Wistar strain.

## Discussion

We found that SHR are resistant to catalepsy, spontaneous locomotion and oral movement impairments in a reserpine-induced progressive animal model of PD. As expected, reserpine-treated Wistar rats presented a progressive increase in the latency to step down the catalepsy bar, which was significant from the 8th injection onwards (Figure 1B). Nevertheless, the SHR reserpine-treated rats presented no increase in the latency to stepdown whatsoever. Thus, the SHR strain was resistant to the difficulty to start a movement induced by the low-dose reserpine treatment. As follows, the same profile of motor impairment was evident in the spontaneous locomotion in the open field. Despite both strains significantly reduced locomotion and speed throughout sessions due to habituation, only reserpine-treated Wistar rats presented impaired spontaneous locomotion 48 h after the 15th injection—to both total distance traveled and time in speed above 0.05 m/s (Figures 2A,B).

Despite the lower susceptibility to motor impairment in the catalepsy and spontaneous locomotion test, reserpine-treated SHR rats presented some extent of oral dyskinesia. However, SHR-RES group presented an increase in oral movements only after the 10th injection, which remained until the 10th day of withdraw. Conversely, WIS-RES group displayed the motor impairment after the 6th injection until the 15th day of withdraw (Figures 3A,B). Moreover, the magnitude of effects on oral movements was higher in Wistar rats than in SHR rats. In summary, the motor profile of reserpine-treated Wistar rats corroborates previous studies (Fernandes et al., 2012; Santos et al., 2013). Importantly, the present results show that the resistance to the acute high-dose reserpine-induced oral dyskinesia that was previously reported (Queiroz et al., 1998; Abílio et al., 2004) was extended to the low-dose repeated regimen and to other motor parameters (catalepsy and spontaneous activity) in the SHR strain.

Regarding the neurochemical analysis, we found a progressive reduction in TH staining in the SNpc that was significant only to WIS-RESw/d group, despite a tendency towards reduction in WIS-RESt (*p* = 0.069; Figure 4A). Oppositely, the reserpine treatment did not result in any reduction of TH staining in the SNpc of SHR rats. On the other hand, both strains presented a reduction in the TH staining after treatment with reserpine in the dSTR, as well as a partial recovery relative to CTR and RESt group in RESw/d groups (Figure 4B). The same profile of recovery was reported for Wistar rats (10 injections of 0.1 mg/kg every other day) by Santos et al. (2013) and Swiss mice (four injections of 1 mg/kg every other day) by de Freitas et al. (2016), which found partial recovery of immunoreactivity for TH in the dSTR after 30 and 60 days of reserpine withdrawn, respectively. These findings suggest a path of progression of the reserpine-induced TH decrement from the fibers (dSTR) to the nucleus (SNpc). Studies with other drug-induced models or parkinsonism also reported a retrograde profile of progression (Zhu et al., 2004; Korecka et al., 2013). Importantly, this trend of progression corroborates contemporary evidence that during early PD progression the dopaminergic loss begins in the projection sites, and subsequently advances to the nucleus (Cheng et al., 2010).

Regarding the present results, the fact that the TH decrement was found in the dSTR but not in the SNpc of SHR reinforces the notion that the dopaminergic loss did not progress in this strain in the same magnitude as it did in Wistar animals. Indeed, the reduction in TH staining in the dSTR of SHR strain was not enough to cause expressive motor impairments. Thus, it is likely that the impairing effects on motor activity of reserpine treatment requires the decrement of TH in the SNpc, which occurred only in the Wistar strain (Figures 4A,B). That is in accordance with the motor progression of PD, because the motor impairments in patients are observed only after the loss of at least a quarter of neurons in the SNpc (Arkadir et al., 2014). Nevertheless, WIS-RESw/d group, despite reduced TH+ neuron count in the SNpc, presented recovery of motor impairment (Figure 4A). Thus, other adaptive mechanisms could be related to this finding. As far as the SHR strain is concerned, other neurochemical alterations—than the TH expression—could have precluded the motor deficit induced by reserpine repeated treatment. Recently, we have found that the magnitude of motor deficit corresponded to the extent of dopamine depletion in Wistar compared to SHR animals (unpublished data).

In this respect, such neurochemical findings could be secondary to alterations in catecholamine levels and metabolism imbalances. Indeed, catecholamines regulate TH activity and expression in the cytoplasm (Dickson and Briggs, 2013). Thus, the resulting increase of DA in the cytoplasm by reserpine treatment may reduce the TH activity in the nigrostriatal pathway. Alternatively, NO and nitrite formation in the cytoplasm also regulates the expression of TH (Daubner et al., 2011; Nakashima et al., 2011). In this respect, reserpine treatment results in oxidative stress and accumulation of reactive oxygen and nitrogen specimens, which ultimately may result in the decrement of TH expression (Arora et al., 2011; Arora and Chopra, 2013; Santos et al., 2013). Here we report reduced lipid peroxidation (evaluated by MDA formation) in SHR relative to Wistar rats. Further, reserpine treatment resulted in increased MDA formation only in the Wistar strain (Figure 6), corroborating findings by Abílio et al. (2004) and Fernandes et al. (2012). That is, SHR rats have a reduced baseline for the formation of oxidative stress products, which ultimately may confer resistance to reserpine-induced oxidative stress. In fact, Abílio et al. (2004) have associated such resistance to increased activity of catalase in the SHR strain. Further investigations of which phosphorylated site of TH is involved in the regulation of TH expression in response to the treatment with reserpine is required to clarify the mechanisms of TH downregulation. Likewise, epigenetic studies could also help to clarify how such cellular oxidative imbalance would modulate the expression of TH (Yang et al., 2011).

From another standpoint, the present results also showed strain differences in α-syn expression. Specifically, both RES-treated groups presented an increase in the expression of α-syn relative to CTR and RESw/d groups (particularly in the dSTR, Figures 4C,D). However, SHR rats presented a reduction of α-syn expression relative to WIS rats in both SNpc and dSTR (Figures 4C,D), irrespective of treatment. This findings corroborate the study of Chiavegatto et al. (2009) that report reduced expression of α-syn in the hippocampus of SHR rats compared to the Lewis strain.

Thus, the reported reduced expression of α-syn in this strain may reflect a significant relevance to the resistance to motor impairments showed by SHR animals. It is well known that the stable form of α-syn is localized in membranes and vesicles of presynaptic terminals in a stable membrane-bound state (Burré, 2015). The stable form is in dynamic equilibrium with the soluble cytosolic α-syn, a monomeric and natively unfolded protein that can be converted into β-sheet containing oligomers (protofibrils), which eventually form amyloid-like fibrils and Lewy bodies (Burré et al., 2013). Thus, an increase in the soluble cytosolic form of α-syn is detrimental to catecholaminergic neurons because there is an increased probability of formation of β-sheet and protofibrils. Nevertheless, despite the increase in α-syn immunostaining, we did not detect any protein inclusions or fibrils formation with the length of repeated reserpine treatment used here (Figure 5). Even so, the reduced α-syn expression in SHR compared to Wistar strain (Figures 4C,D) denotes a mechanism by which reserpine-treated SHR rats may cope with DA imbalances and TH downregulation.

Indeed, α-syn is involved in the homeostasis of DA by inhibiting the expression and activity of TH and VMAT2 (Baptista et al., 2003; Guo et al., 2008), and interrupting dopamine balance by causing increased cytosolic dopamine levels (Perez et al., 2002). Conversely, catecholamine metabolites, such as 5-S-Cysteinyldopamine, modulate α-syn expression through oxidative stress (Aureli et al., 2014). Thus, reserpine-induced increases in catecholamine metabolites and oxidative imbalances may be in the core of the neurochemical alterations reported here (Leão et al., 2015). Accordingly, SHR rats, which show decreased imunostainning for α-syn (Figure 4D), also presented reduced levels of lipid peroxidation (Figure 6). Therefore, SHR rats appear to be better suited to cope with oxidative imbalances resulting from reserpine treatment, and this feature could be related to the different profiles of TH and α-syn expressions between Wistar and SHR strains.

Of notice, irrespective of strains differences, this is the first study to show increased expression of α-syn in reserpine-treated mammals. To our knowledge, only one previous study investigated the effects of reserpine on α-syn expression in a *Droshophila melanogaster* model. That study reported inhibition of the autophagic flux, disruption in protein degradation, and accumulation of α-syn-rich protein aggregates (Lee et al., 2015). Accordingly, VMAT2-deficient mice presented age-dependent neurodegeneration in the SNpc, followed by α-syn accumulation and TH immunostaining reduction (Caudle et al., 2007; Taylor et al., 2009). Despite overall motor and neurochemical results pointing towards a reversible reserpine-induced insult to the nigrostriatal pathway (present results; Fernandes et al., 2012; Santos et al., 2013), we do not discard some extent of neurodegeneration and α-syn rich protein inclusions or fibrils in longer treatment regimens. As follows, long-term VMAT2 blockade or loss of function results in irreversible neurochemical and behavioral alterations (Neisewander et al., 1991), as well as neurodegeneration (Taylor et al., 2011). Thus, investigation of neurodegenerative markers and defective protein accumulation are still required to clarify this matter. For instance, reserpine treatment results in increased expression of caspase-3 (Arora and Chopra, 2013; Liu et al., 2014) and reduction of Bcl-2 (Liu et al., 2014; El-Ghazaly et al., 2015), which are important markers of apoptotic pathways commitment. Finally, the increase in the α-syn found in reserpine-treated animals is a putative secondary mechanism by which TH is downregulated. Indeed, α-syn reduces TH expression through inhibition of phosphorylation at Ser40 of TH (Peng et al., 2005; Wu et al., 2011).

In summary, even though we do not report any sign of α-syn positive protein inclusions, we suggest that the behavioral and neurochemical alterations here reported resembles a closer relationship to early PD molecular alterations. Nonetheless, other chemically induced PD models do not account for this histopathological hallmark (Meredith et al., 2008; Blesa et al., 2012). Importantly, the differences between the two strains could shed light on these initial cellular alterations. That is, the different profiles described here may result from compensatory and/or plastic mechanisms related to the reduced α-syn expression, resistance to the TH decrement in the SNpc, and reduced oxidative damage exhibited by the SHR strain.

In view of such findings, it is clear that the interaction between the genetic background of rat strains (genetic factors) and neurotoxic treatments (environmental factors) is substantial to the expression of motor and cellular alterations in the progressive PD model induced by repeated reserpine treatment. Beyond the aforementioned evidence, SHR rats present other cellular features of the dopaminergic system that are relevant to the understanding of PD. For example, SHR rats express more D1 and DAT in the striatum (Watanabe et al., 1997), but present reduced function of the latter (Russell, 2003). They also seem to express less TH in the pre-frontal cortex (King et al., 2000) among other neurochemical alterations (for a review see Viggiano et al., 2004). Ideally, the investigation of the SHR strain neurochemical differences in the catecholaminergic system should be extended to other parameters and rat strains.

In conclusion, here we demonstrate that SHR rats are resistant to a progressive reserpine-induced PD model. In the Wistar strain, the motor impairments were followed by compatible neurochemical alterations that reflected PD progression, namely reduced TH and increased α-syn expressions in the substantia nigra and dSTR. On the other hand, motor and neurochemical data from the SHR strain revealed a dissociation of neurochemical alterations and motor behavior—for example, reduced TH expression in the dSTR, but no motor impairment. Here we highlight some of the neurochemical differences between these strains that may be adaptive to PD resistance, such as reduced expression of α-syn and oxidative stress products in the SHR strain. Nevertheless, the underlying mechanism by which such resistance is granted to SHR rats remains to be investigated. In view of these findings, we suggest the SHR resistant strain and the repeated low-dose reserpine protocol as valuable animal resources to investigate the relevance of genetic, cellular and behavioral features to the of progression of PD.

## Author Contributions

AHFFL, GSI, AMR and RHS designed the study. AHFFL, YSRM, AFS, AMM, CLCC and AMR performed the experiments. AHFFL analyzed data and wrote the manuscript. VCA, RCGKE, JSC and AMR contributed with theoretical discussions and technical insights. RHS contributed in analysis and writing, and revised the final version.

## Conflict of Interest Statement

The authors declare that the research was conducted in the absence of any commercial or financial relationships that could be construed as a potential conflict of interest.
